# Crosstalk between Desmoglein 2 and Patched 1 accelerates chemical-induced skin tumorigenesis

**DOI:** 10.18632/oncotarget.3309

**Published:** 2015-03-24

**Authors:** Donna M. Brennan-Crispi, Claudia Hossain, Joya Sahu, Mary Brady, Natalia A. Riobo, Mỹ G. Mahoney

**Affiliations:** ^1^ Department of Dermatology and Cutaneous Biology, Thomas Jefferson University, Philadelphia, PA, USA; ^2^ Department of Biochemistry and Molecular Biology, Thomas Jefferson University, Philadelphia, PA, USA; ^3^ Sidney Kimmel Cancer Center, Thomas Jefferson University, Philadelphia, PA, USA

**Keywords:** Desmoglein 2, Hedgehog Signaling, Patched 1, Basal Cell Carcinoma, Squamous Cell Carcinoma

## Abstract

Aberrant activation of Hedgehog (Hh) signaling is causative of BCCs and has been associated with a fraction of SCCs. Desmoglein 2 (Dsg2) is an adhesion protein that is upregulated in many cancers and overexpression of Dsg2 in the epidermis renders mice more susceptible to squamous-derived neoplasia. Here we examined a potential crosstalk between Dsg2 and Hh signaling in skin tumorigenesis. Our findings show that Dsg2 modulates Gli1 expression, *in vitro* and *in vivo*. Ectopic expression of Dsg2 on Ptc1^+/lacZ^ background enhanced epidermal proliferation and interfollicular activation of the Hh pathway. Furthermore, in response to DMBA/TPA, the Dsg2/Ptc1^+/lacZ^ mice developed squamous lessons earlier than the WT, Ptc1^+/lacZ^, and Inv-Dsg2 littermates. Additionally, DMBA/TPA induced BCC formation in all mice harboring the Ptc1^+/lacZ^ gene and the presence of Dsg2 in Dsg2/Ptc1^+/lacZ^ mice doubled the BCC tumor burden. Reporter analysis revealed activation of the Hh pathway in the BCC tumors. However, in the SCCs we observed Hh activity only in the underlying dermis of the tumors. Furthermore, Dsg2/Ptc1^+/lacZ^ mice demonstrated enhanced MEK/Erk1/2 activation within the tumors and expression of Shh in the dermis. In summary, our results demonstrate that Dsg2 modulates Hh signaling, and this synergy may accelerate skin tumor development by different mechanisms.

## INTRODUCTION

Desmosomes are multi-protein, cell-cell adhesion complexes critical for tissue integrity and function [1, 2]. Desmoglein 2 (Dsg2), the most ubiquitously expressed desmosomal cadherin, is found in all simple epithelia and select non-epithelial cells such as cardiomyocytes [3–5]. In the skin, Dsg2 is highly expressed in the hair follicles but is found only at a low level in the basal layer of the interfollicular epidermis [6]. The role of Dsg2 in tumorigenesis emerged with the findings that Dsg2 expression is highly upregulated in several epithelial-derived malignancies including basal cell carcinomas (BCC) and squamous cell carcinomas (SCC) [6]. Furthermore, we recently reported that ectopic expression of Dsg2 in the superficial epidermis renders mice more susceptible to tumor development [7].

The role of desmogleins in cell-cell adhesion has been well established [9]. However, recent work has recognized additional functions of desmogleins beyond the desmosomes. We previously demonstrated that Dsg2 enhances keratinocyte proliferation and survival, and activates mitogenic signaling cascades, including the MEK/Erk, PI3K/Akt and JAK/Stat3 pathways [7]. Furthermore, we established that Dsg2 interacts with caveolin-1, a key regulator of cell signaling [8]. Additionally, knockdown of Dsg2 in colon cancer cells inhibits proliferation by suppressing EGFR signaling [9]. Taken together, these data support the notion that Dsg2 may influence cancer development and progression, in part by regulating key signaling pathways that maintain skin homeostasis.

The Hedgehog (Hh) signaling pathway is another crucial regulator of skin homeostasis. Aberrant activation of the pathway in the skin is the causative factor of BCC, both sporadic and familiar [12]. The hallmark of canonical activity is the activation of the Gli family of transcription factors [13]. Gli activation is negatively regulated by the tumor suppressor Patched 1 (Ptc1). In the absence of the Hh ligands, Sonic (Shh), Indian (Ihh) or Desert (Dhh) Hh, Ptc1 inhibits Smoothened (Smo), leading to processing of Gli2 and Gli3 to their repressor forms, which blocks target gene transcription. The Hh pathway is activated upon binding of a Hh ligand to Ptc1, which leads to derepression of Smo and activation of the Gli transcription factors. Importantly, both Gli1 and Ptc1 are targets of Gli transcription and serve as readouts of the Hh pathway.

In the normal skin, Hh signaling is high during specific stages of the hair follicle cycle, but undetectable in the interfollicular epidermis [14]. However, the Hh pathway is upregulated in all BCCs, either by loss of function of Ptc1 (PTCH1 in humans) or gain of function mutations of Smo [12]. Gorlin's syndrome, a condition in which patients develop hundreds of BCCs, is caused by PTCH1 haploinsufficiency, typically followed by subsequent somatic loss of heterozygosity (LOH) due to insults like UV irradiation [15]. The role of Hh signaling in SCCs is not well-defined, with only certain subsets displaying markers of Hh activation [16], though the Ptc1^+/lacZ^ mouse model has shown increased susceptibility to UV-induced SCC formation [10]. This pattern of widespread upregulation of Hh signaling markers in BCCs and heterogeneous activation in SCCs is reminiscent of Dsg2. Interestingly, a number of cell signaling pathways regulated by Dsg2, including PI3K/Akt, MEK/Erk1/2 and JAK/Stat3, can synergize with the Hh pathway to stimulate Gli transcriptional activity [11, 12]. Based on this evidence, we proposed to determine if Dsg2 and Hh signaling cooperate in skin tumor development.

To explore the interaction between Dsg2 and Hh signaling we crossed two established animal models: Ptc1^+/lacZ^ and Inv-Dsg2 mice. Our Inv-Dsg2 transgenic animals, which overexpress Dsg2 in the suprabasal layers of the epidermis under control of the involucrin promoter [8], are an established model for Dsg2-mediated squamous tumor development. The Ptc1^+/lacZ^ mice are heterozygous for Ptc1, with one copy replaced with a functional *lacZ* gene that serves as reporter of pathway activation, since Ptc1 is a Gli-target gene. Though generated as a mouse model for Gorlin syndrome; unlike humans, these mice only rarely develop spontaneous BCC [13] with advanced age, but require additional hits such as UV or γ-irradiation which can induce loss of p53 [14], or to be bred into a p53 null background to develop significant skin tumors [10]. Thus, the Ptc1^+/lacZ^ mice serve primarily as reporters of Hh pathway activity, but are primed for skin tumor formation. Therefore, we crossed these two mouse models to determine if Dsg2 and Hh interact during chemical-induced tumor development. Our results suggest a synergistic interaction between Dsg2 and Hh signaling in the development of both SCCs and BCCs.

## RESULTS

### Dsg2 enhances canonical Hh signaling in mouse skin and in cultured keratinocytes

**Figure 1:**
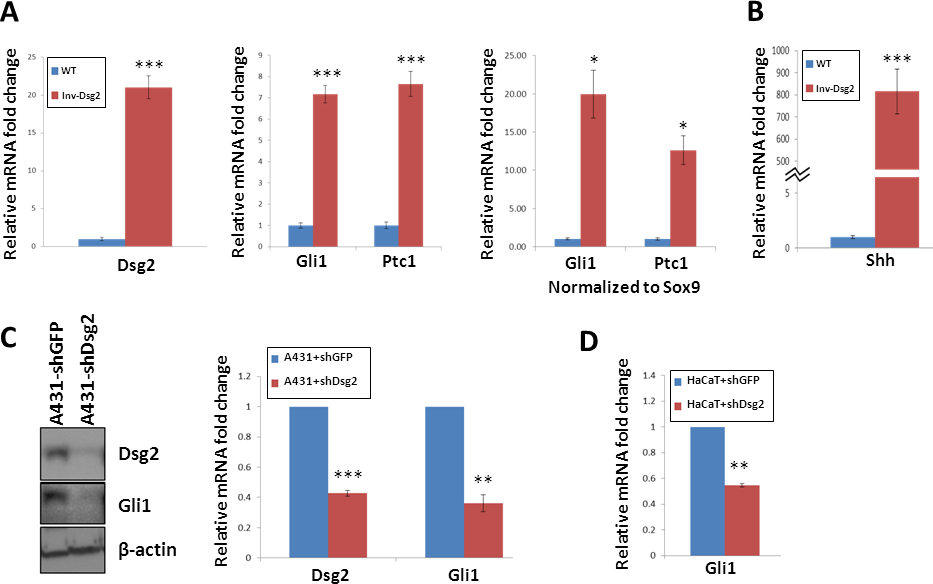
Dsg2 enhances Gli1 expression **A–B.** qPCR analysis of 6-week old backskin from Inv-Dsg2 (*n* = 8) and WT (*n* = 6) mice. Samples normalized to internal control (GAPDH); in addition to control for follicles in the anagen stage of development, samples were normalized to Sox9. **C.** Western analysis of A431-shDsg2 and A431-shDsg2 lysates (*n* = 3), for Dsg2 and Gli1. Actin serves as a loading control. Densitometry measurements were completed using image J. **D.** qPCR of HaCaT-shDsg2 and HaCaT-shGFP control cells (*n* = 3). Samples were normalized to GAPDH. All data are shown as the mean ± SEM; *p < 0.05, **p < 0.01, ***p < 0.001; *t*-test.

To determine the effect of Dsg2 on Hh signaling *in vivo*, we quantified expression of *gli1* and *ptc1* mRNA in the skin of 6 week-old Inv-Dsg2 transgenic mice and wild type (WT) littermates. Analysis by qPCR confirmed enhanced Dsg2 expression and revealed an ~7-fold increase in *gli1* and *ptc1* mRNA in the skin of Inv-Dsg2 mice (Figure 1A). As activation of the Hh pathway is a hallmark of hair follicles in anagen, we also normalized the *gli1* and *ptc1* Ct values to the anagen hair follicle marker *sox9* to confirm that the increase in Hh target genes was not secondary to an increased number of anagen follicles. Strikingly, normalization of *gli1* and *ptc1* to *sox9* resulted in an even higher upregulation of *gli1* and *ptc1* (~20 and 10-fold respectively) (Figure 1A); suggesting that the observed increase in *gli1* and *ptc1* could not be accounted for by an increase in hair follicle number or an alteration in the hair follicle cycle. Remarkably, expression of Shh at the transcript level was highly upregulated in the skin of Inv-Dsg2 mice (800-fold vs. WT), suggesting that it may account for increased Gli target gene expression *in vivo* (Figure 1B). Thus, overexpressing Dsg2 in the epidermis increases canonical Hh pathway activity *in vivo*.

To obtain further proof that Dsg2 levels regulate activation of the canonical Hh pathway, we utilized SCC-derived A431 cells stably expressing control (A431+shGFP) or Dsg2 (A431+shDsg2) short hairpin RNA [21]. Stable knockdown of Dsg2 was accompanied by a striking reduction of Gli1 (Figure 1C), suggesting that Dsg2 might stimulate canonical Hh signaling. To test whether this holds true in non-tumorigenic keratinocytes, we compared expression the Hh target genes *gli1* and *ptc1* in HaCaT cells transfected with shRNA to GFP (HaCaT+shGFP) or Dsg2 (HaCaT+shDsg2). Because HaCaT cells express very low levels of Gli1, that are not detectable at the protein level, only qPCR was used. Knockdown of Dsg2 resulted in a significant 50% decrease in *gli1* transcript levels compared to controls (Figure 1D). Altogether, these observations demonstrate that Dsg2 promotes canonical Hh signaling both *in vitro* and *in vivo*.

### Dsg2-mediated hyperproliferation is enhanced on the Ptc1^+/lacZ^ background

**Figure 2:**
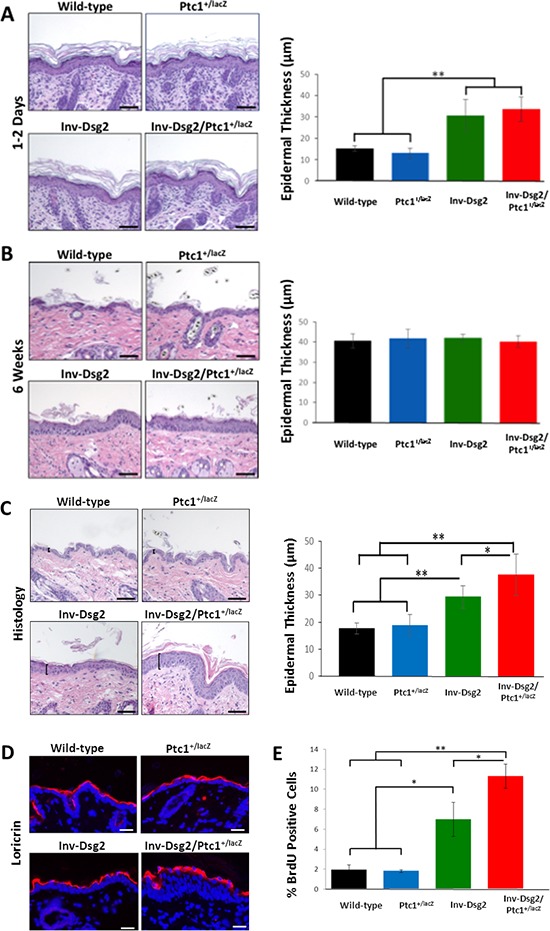
Inv-Dsg2/Ptc1^+/lacZ^ mice exhibit epidermal hyperproliferation **(A–C)** Histological analysis of newborn mouse skin (A) 6 weeks (B) and 3-month (C) mouse backskin. Epidermal thickness was measured as indicated by brackets. Graphical representation of average epidermal thickness is presented in microns. Newborn: WT (*n* = 3); Ptc1^+/lacZ^ (*n* = 4); Inv-Dsg2 (*n* = 6); Inv-Dsg2/Ptc1^+/lacZ^ (*n* = 4); 6 week: WT (*n* = 5); Ptc1^+/lacZ^ (*n* = 2); Inv-Dsg2 (*n* = 5); Inv-Dsg2/Ptc1^+/lacZ^ (*n* = 5); 3 month: WT (*n* = 10); Ptc1^+/lacZ^ (*n* = 6); Inv-Dsg2 (*n* = 4); Inv-Dsg2/Ptc1^+/lacZ^ (*n* = 7). **D.** IF of the cornified envelop marker loricrin (red); nuclei counterstained with DAPI (blue). **E.** Graphical representation of number of BrdU positive cells per 100 basal keratinocytes. WT (*n* = 5); Ptc1^+/lacZ^ (*n* = 3); Inv-Dsg2 (*n* = 3); Inv-Dsg2/Ptc1^+/lacZ^ (*n* = 4). The data are shown as the mean ± SEM. *p < 0.05; **p < 0.01; *t*-test. Scale bar = 50 microns.

To assess the biological consequence of the crosstalk between Dsg2 and Hh signaling, we bred our Inv-Dsg2 mice and the Ptc1^+/lacZ^ reporter mice to generate Inv-Dsg2/Ptc1^+/lacZ^ compound animals, single transgenic (Inv-Dsg2 and Ptc1^+/LacZ^) and WT littermates, which were born at the expected Mendelian rates. We measured epidermal thickness of the mice at different developmental stages (1–2 days, 6 weeks, and 3 months of age). Newborn mice appeared normal, with all genotypes exhibiting comparable epidermal thickness (Figure 2A). As we published before, by 6 weeks the Inv-Dsg2 mice showed signs of hyperplasia (Figure 2B). Strikingly, by 3 months of age the Inv-Dsg2/Ptc1^+/lacZ^ compound mice exhibited statistically significant increased hyperplasia compared to their Inv-Dsg2 counterparts, while there was no difference in average epidermal thickness between WT and Ptc1^+/lacZ^ mice (Figure 2C), suggesting that a Ptc1 haploinsufficiency allows for enhanced effects Dsg2 on skin homeostasis.

Next, we sought to determine if the increased hyperplasia resulted from defective terminal differentiation or enhanced proliferation. To assess significant defects on terminal differentiation, we analyzed the expression of cornified envelope markers. Immunofluorescent staining of loricrin revealed normal staining in the epidermis in all mice (Figure 2D). Additional cornified envelope markers, involucrin and filaggrin displayed similar results (data not shown), suggesting that the terminal differentiation proceeds relatively normally. While we cannot rule out more subtle effects on differentiation, this data suggests that impairment of terminal differentiation could not explain the enhanced skin hyperplasia of Dsg2/Ptc1^+/lacZ^ mice. In contrast, BrdU incorporation revealed increased proliferation in the basal layer of the skin of Inv-Dsg2/Ptc1^+/lacZ^ mice compared to Inv-Dsg2 and Ptc1^+/lacZ^ or WT animals (Figure 2E). These results indicate that Dsg2-mediated epidermal proliferation is enhanced on the Ptc1^+/lacZ^ background, and suggests a possible synergistic effect of the two signaling pathways, as there is no change in Ptc1^+/lacZ^ animals compared to wild-type.

### Hh signaling is activated in the interfollicular epidermis of Inv-Dsg2/Ptc1^+/lacZ^ mice

**Figure 3:**
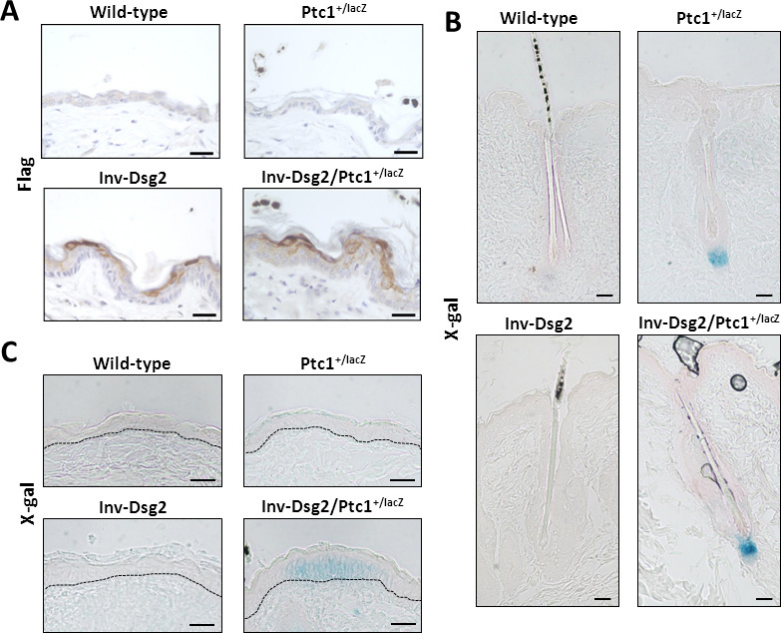
Inv-Dsg2/Ptc1^+/lacZ^ mice exhibit interfollicular activation of the Hh pathway **A.** IHC of mouse backskin with anti-Flag antibodies detects Flag-tagged Dsg2 transgene expression (brown) in superficial epidermis, nuclei counterstained with hematoxylin. **(B–C)** X-Gal staining of mouse backskin depicting β-gal expression/Hh activity (blue) and counterstained with nuclear fast red, hair follicles which serve as positive controls **B.** and interfolicular epidermis **C.** Scale bar = 25 microns. Dashed line demarcates basement membrane zone.

To investigate the origin of the phenotypic differences between the Inv-Dsg2 and Ptc1^+/lacZ^ crossed progeny, we evaluated the expression of the Flag-tagged Dsg2 transgene and Hh pathway activity in the skin of all genotypes and control WT mice at three months of age. As expected, Dsg2.Flag expression was only detected by immunohistochemistry in Inv-Dsg2 and Inv-Dsg2/Ptc1^+/lacZ^ mice and was restricted to the suprabasal epidermis (Figure 3A). The X-gal staining which reports expression of Ptc1-lacZ, a marker of canonical Hh pathway activation, was detected only in mice harboring the lacZ reporter gene. Both Ptc1^+/lacZ^ and Inv-Dsg2/Ptc1^+/lacZ^ mice exhibited a comparable activation of the Hh pathway in the dermal papilla of hair follicles (Figure 3B). Differences between Ptc1^+/lacZ^ and Inv-Dsg2/Ptc1^+/lacZ^ animals emerged when the interfollicular epidermis (IFE) was examined. The IFE is typically void of detectable Hh pathway activity, and indeed Ptc1^+/lacZ^ mice showed little to no X-gal staining outside of the hair follicles. Increased Hh reporter activity was observed in localized regions of the IFE of the Inv-Dsg2/Ptc1^+/lacZ^ mice (Figure 3C), indicating that Dsg2 overexpression stimulates Hh signaling in keratinocytes *in vivo*.

### Dsg2/Ptc1^+/lacZ^ mice exhibit accelerated squamous tumor development

**Figure 4:**
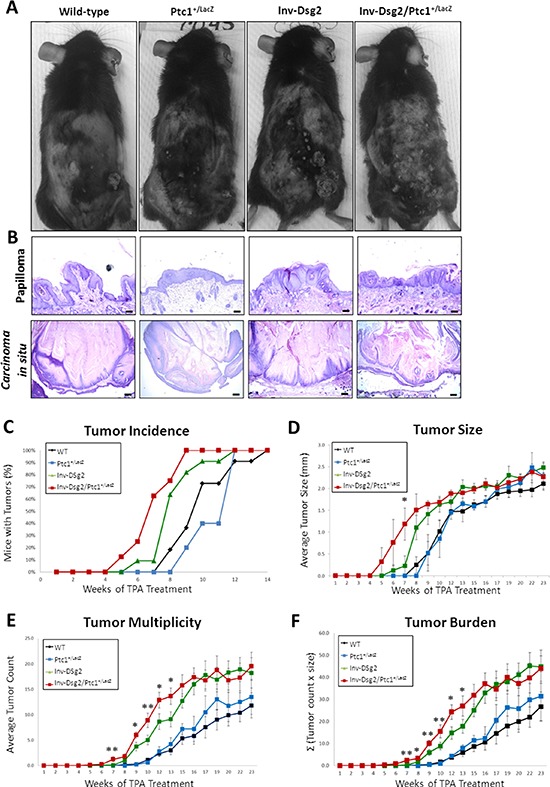
DMBA-TPA treatment results in accelerated growth of squamous derived neoplasia in Inv-Dsg2/Ptc1^+/LacZ^ mice **A.** Macroscopic tumor formation is observed in all mice after DMBA-TPA treatment. **B.** Histological analysis reveals papillomas and *in situ* carcinomas in all genotypes (scale bars = 100 and 250 microns, respectively). **C.** Tumor incidence is depicted as percentage of mice exhibiting tumors. **D.** The average tumor diameter was determined for all mice. **E.** Tumor multiplicity represents the average number of tumors per genotype. **F.** Tumor burden, accounts for size and number of tumors, is represented as the sum of tumors x size (Σ). WT (*n* = 11); Ptc1^+/lacZ^ (*n* = 5); Inv-Dsg2 (*n* = 10); Inv-Dsg2/Ptc1^+/lacZ^ (*n* = 8). The data are shown as the mean ± SEM. *p < 0.05; **p < 0.01; *t*-test.

We previously demonstrated that the Inv-Dsg2 mice were more susceptible to chemical-induced squamous tumor development [7]. Since compound Inv-Dsg2/Ptc1^+/lacZ^ mice showed increased Hh signaling and hyperproliferation compared to Inv-Dsg2 animals, we investigated whether they are more susceptible to DMBA-TPA induced tumor development. Mice were treated once with DMBA, followed by twice-weekly administrations of TPA for 26 weeks. All genotypes developed macroscopic lesions (Figure 4A) by 14 weeks of TPA treatment; however, tumors in the Inv-Dsg2/Ptc1^+/lacZ^ mice emerged as early as 5 weeks of TPA treatment, and reached 100% incidence by week 9 (Figure 4C). In comparison, the other three genotypes developed tumors around week 7–8 of treatment and did not reach 100% incidence until weeks 12–14 (Figure 4C). Thus, the Inv-Dsg2/Ptc1^+/lacZ^ mice have accelerated macroscopic tumor formation compared to all other genotypes.

To determine if there were any differences in tumor load, we quantified the average size of tumors, the number of tumors per animal (tumor multiplicity) and the average tumor burden in all four genotypes. Tumor development in WT and Ptc1^+/lacZ^ mice was highly comparable, suggesting no significant differences between the two genotypes throughout the duration of treatment. As we previously reported, mice overexpressing Dsg2 developed more and larger tumors than WT mice. Comparison of Inv-Dsg2 Inv-Dsg2/Ptc1^+/lacZ^ mice revealed no significant difference in tumor size (Figure 4D). However, Inv-Dsg2/Ptc1^+/lacZ^ mice developed significantly more tumors than the Inv-Dsg2 counterparts during the early stages of development, weeks 7–13 (Figure 4E). Interestingly, after week 13 the difference in tumor number between Inv-Dsg2 and Inv-Dsg2/Ptc1^+/lacZ^ mice leveled off. Similarly, average tumor burden, which takes into account tumor number and size, showed no difference between Inv-Dsg2 and Inv-Dsg2/Ptc1^+/lacZ^ mice after week 13 (Figure 4F). Lack of difference in overall tumor burden but evidence of decreased tumor latency in response to chemical carcinogens in Inv-Dsg2/Ptc1^+/lacZ^ mice, suggests a possible synergistic effect of the two pathways on the rate of tumor promotion.

**Figure 5:**
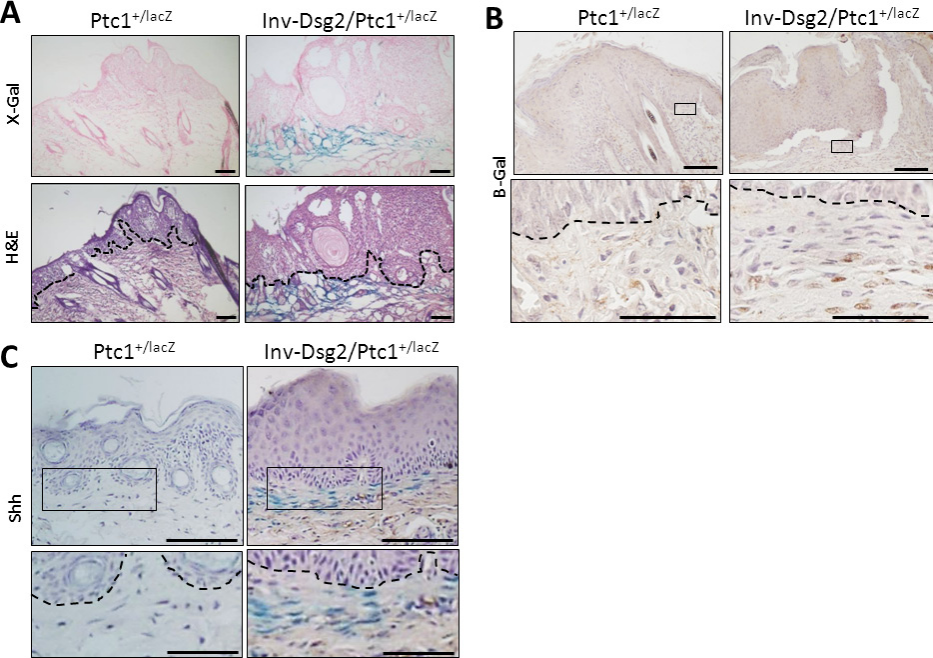
Inv-Dsg2/Ptc1^+/lacZ^ mice display Hh activity in the tumor stroma **A.** Consecutive sections of papillomas reveal the tumor epithelium is negative for X-gal, but tumor stroma of the Inv-Dsg2/Ptc1^+/lacZ^ mice positively stained for Hh activation (blue). **B.** β-gal IHC confirms activation of the Hh pathway in fibroblasts of the tumor stroma. **C.** IHC staining reveals Shh in the SCC tumor stroma. Dashed line demarcates basement membrane zone. Scale bar = 100 microns, 50 microns for insets.

**Figure 6:**
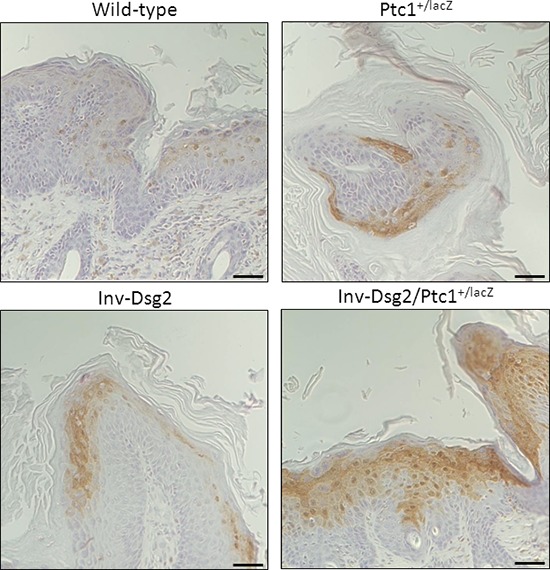
Inv-Dsg2/Ptc1^+/lacZ^ squamous lesions exhibit enhanced activation of Phospho-Erk1/2 IHC of Phospho-Erk1/2 reveals increased nuclear localization within the Inv-Dsg2/Ptc1^+/lacZ^ squamous lesions. Note that signal is not observed in BCC. Scale bar = 100 microns, 50 microns for insets. Scale bar = 100 microns.

We also compared the tumor morphology at the end of the treatment protocol. All genotypes developed histologically similar papillomas and *in situ* carcinomas (Figure 4B). None of the animals developed full-blown SCC or showed signs of metastasis. Surprisingly, yet in agreement with the similar burden and morphology, neither X-gal nor β-gal staining revealed activation of the Hh pathway in squamous tumors of Ptc^1+/lacZ^ or Inv-Dsg2/Ptc1^+/lacZ^ mice (Figure 5). However we detected strong X-gal staining in the dermis underlying the tumors in Inv-Dsg2/Ptc^1+/lacZ^ mice, which was not observed in Ptc1^+/lacZ^ animals (Figure 5A). β-gal staining further confirmed the activation of the Hh pathway in the dermal fibroblasts of the compound mice (Figure 5B). We also found an increased expression of Shh by immunohistochemical (IHC) staining in the dermis, but not in the epidermis, of Dsg2 transgenic mice, which explains the X-gal and β-gal staining of dermal fibroblasts (Figure 5C). It is therefore possible that activation of the Hh pathway in the dermis in a Shh-dependent manner could account for the earlier tumor emergence in the Inv-Dsg2/Ptc1^+/lacZ^ mice. Finally, to determine if there is a change in growth and survival signaling in the SCC tumors of all genotypes, we stained for phosphorylated Erk1/2. Interestingly, while tumors of all genotypes exhibit some level of staining, total phosphorylation and nuclear localization were more prominent in the Inv-Dsg2/Ptc1^+/lacZ^ mice than in any other genotype (Figure 6), as confirmed by confirmed by four independent blind observers.

### Dsg2 enhances BCC formation in Ptc1 heterozygote animals

**Figure 7:**
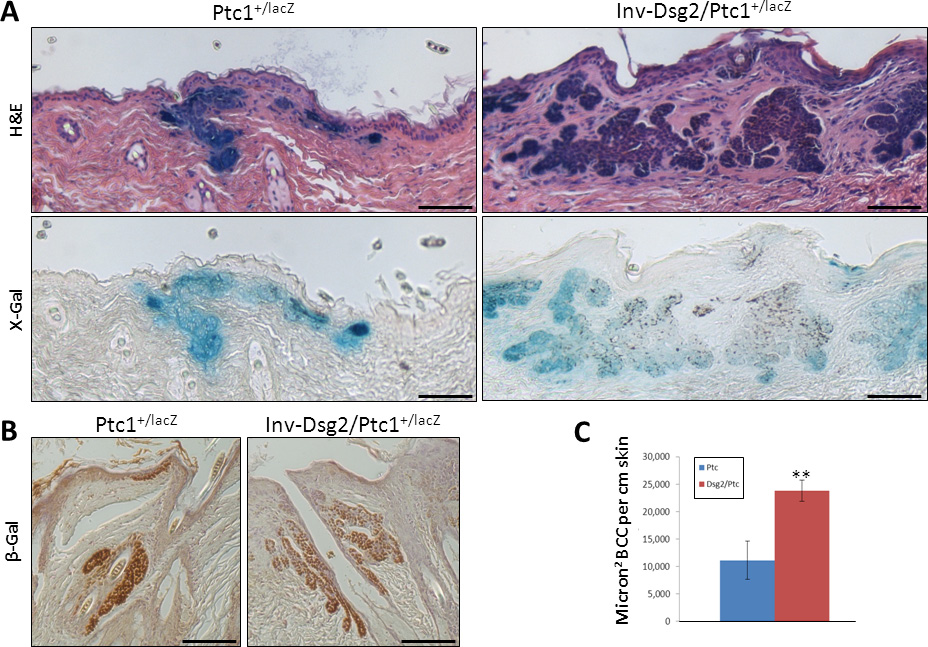
DMBA-TPA induces enhanced BCC formation in Inv-Dsg2/Ptc1^+/LacZ^ mice **A.** Microscopic BCC formation is observed in all mice harboring the Ptc1^+/lacZ^ gene (H&E), and these tumors display characteristic activation of the Hh pathway (X-gal). BCCs were not found in any WT or Inv-Dsg2 mice. **B.** β-gal IHC confirms activation of the Hh pathway in the induced BCCs. Scale bars = 100 microns. **C.** BCC tumor burden is increased more than two-fold in Inv-Dsg2/Ptc1^+/LacZ^ mice. Ptc1^+/lacZ^ (*n* = 4); Inv-Dsg2/Ptc1^+/lacZ^ (*n* = 6). The data are shown as the mean ± SEM. **p < 0.01; *t*-test.

Remarkably, while performing the pathology analysis, we unexpectedly observed BCC tumor development in response to DMBA-TPA in Ptc1^+/lacZ^ and Dsg2/Ptc1^+/lacZ^ mice but not in WT or Inv-Dsg2 animals. BCCs in both Ptc1^+/lacZ^ and Inv-Dsg2/Ptc1^+/lacZ^ mice had a similar morphology (Figure 7A) and exhibited classical activation of the Hh pathway, as determined by strong X-gal staining (Figure 7A). IHC staining of nuclear β-gal further confirmed pathway activation in the BCCs (Figure 7B). Importantly, the BCC tumor burden was ~2 fold larger in Inv-Dsg2/Ptc1^+/lacZ^ compound animals compared to Ptc1^+/lacZ^ mice (Figure 7C), suggesting that the presence of Dsg2 enhances BCC development in the Ptc1 heterozygote background.

## DISCUSSION

Emerging evidence supports the existence of non-adhesive functions of Dsg2. We and others have shown that Dsg2 promotes keratinocytes proliferation and tumor development by activation of mitogenic signaling pathways [7, 9]. This work expands on our previous findings that established a role for Dsg2 in skin carcinogenesis, by identifying an unrecognized function for Dsg2 in enhancing canonical Hh signaling.

In the present study we demonstrate the existence of a generalized crosstalk between Dsg2 and Hh signaling in different cell types that promotes epidermal proliferation, accelerates squamous-derived tumorigenesis, and enhances BCC development *in vivo*. Our findings suggest that Dsg2 or its downstream signaling, which is not fully understood, might constitute potential new targets for intervention in squamous and basal cell-derived lesions.

Using compound Inv-Dsg2/Ptc1^+/lacZ^ mice, which overexpress Dsg2 in the suprabasal epidermis and are heterozygous for Ptc1, we showed a remarkable synergism in the promotion of epidermal hyperproliferation and upregulation of the canonical Hh pathway in some regions of the IFE compared to the Ptc1^+/lacZ^ genotype. We hypothesize that these IFE regions with enhanced Hh activity may expand to form either spontaneous or DMBA-TPA induced BCC. BCC can arise in Ptc1 heterozygous mice either through LOH of the WT Ptc1 allele [10] or through activation of the pathway via other means. For instance Ptc1^+/–^ mice crossed with mice Sufu^+/–^, a negative regulator of the Hh pathway downstream of Smo, increased basaloid epidermal proliferations [16]. Additionally, Ptc1^+/lacZ^ mice with loss of p53 function are also more prone to spontaneous BCC formation [17]. Thus, we speculate that the normally effective regulation of the Hh pathway by just a single allele of Ptc1 is overcome, potentially through the inhibition of the function of that single copy of Ptc1 via ligand binding and/or by downstream synergism with other Dsg2-mediated signaling. Furthermore, we observe spontaneous early BCC development only in Inv-Dsg2/Ptc1^+/lacZ^ mice (unpublished observations). While Ptc1^+/lacZ^ mice can develop spontaneous BCCs, they are rare and occur only at more advanced ages. We also note that BCCs are not reported in WT or Inv-Dsg2 mice, even at advanced ages.

Overexpression of Dsg2 in the Ptc1 heterozygous background also leads to accelerated squamous tumor development in response to DMBA/TPA treatment; however, this effect is only observed during early tumor development. This suggests that Dsg2-Hh crosstalk can enhance tumor promotion, but that it is most likely dispensable for Dsg2-mediated cutaneous tumor development and progression. Indeed, this corroborates the human tissue array studies showing that Hh signaling is not required for cutaneous SCC development, but increased Shh expression is associated with decreased survival in oral SCC [18].

Interestingly, while Ptc1^+/lacZ^ mice were also shown to be more prone to SCC development in response to UV-irradiation compared to WT mice, those lesions were also negative for Gli1 and canonical pathway activation [10]. We did not observe a similar increase in response to DMBA-TPA treatment, which typically occurs as a result of Ras mutations [19]. This may suggest differing mechanisms for squamous tumor development, and possibly a non-canonical role for Ptc1 in UV-mediated tumorigenesis.

Surprisingly in our Inv-Dsg2/Ptc1^+/lacZ^ mice, the activation of the Hh pathway is not observed in SCC tumors, but rather in the dermis underlying the tumors. We also observed an increase in Shh localized to the dermis, which may explain the increase canonical Hh pathway activation in these dermal fibroblasts. Hh activation has been reported in the stroma of various tumors [20, 21] and mounting evidence suggests that the stroma plays vital roles in tumor promotion, maintenance, and progression [22]. We propose that the activation of Hh signaling in the dermis facilitates a tumor microenvironment conducive to accelerated growth, similar to recent studies in prostate and pancreatic cancers [23, 24]. Therefore, we hypothesize that Dsg2-mediated squamous tumor promotion may be accelerated by the activation of Hh signaling in the adjacent dermis. Indeed, increased activation of Erk1/2 suggests enhanced growth and survival signaling activity in Inv-Dsg2/Ptc1^+/lacZ^ tumors.

Finally, another potential driver of Dsg2-Hh tumorigenesis is activation of non-canonical Hh signaling [25, 26]. While this study was designed to analyze canonical, Gli-dependent Hh signaling, Ptc1 and Smo exert other functions independent of transcription that regulate cell survival. In particular, Ptc1 is an inducer of apoptosis, and inhibition of its function by Dsg2 could increase cell survival and tumor formation [27]. Future investigations are needed to determine what role Dsg2 may have in activating non-canonical Hh signaling, its role on keratinocyte proliferation and survival.

To our knowledge, this is the first report that a desmosomal cadherin can facilitate activation of the Hh signaling pathway and promote BCC and SCC tumor formation. The significance of Dsg2-Hh crosstalk has implications beyond cutaneous tumors, as both players are deregulated in a variety of cancers including oral head and neck SCC, gastric, and prostate cancers [24, 26, 28–32]. Moreover, our data specifically suggests that two distinct mechanisms of Dsg2-Hh synergy are responsible for the effects on the different tumor types. DMBA/TPA-induced BCCs in Inv-Dsg2/Ptc1^+/lacZ^ mice exhibit classical activation of the Hh pathway; whereas, squamous-derived neoplasia lack detectable Hh activity. Furthermore, unlike BCCs, squamous lesions are accompanied by Shh expression and Hh pathway activity in the underlying dermis, and the tumors themselves harbor increased levels of activated Erk1/2. Future studies to elucidate these differing mechanisms of Hh pathway induction in response to Dsg2 are underway. The implication of the crosstalk in various tumors has the potential to identify targets for novel and tailored cancer treatments. Furthermore, these findings may be relevant to the current therapeutic options for squamous lesions, as they suggest that the canonical Hh pathway active in the dermis adjacent to those tumors has a positive influence in tumor promotion, and could be suggestive of an additional route of intervention using the FDA approved Smo inhibitor Vismodegib.

## MATERIALS AND METHODS

### Ethics statement

Investigation using animals has been conducted in accordance with the ethical standards according to national and international guidelines and has been approved by the authors' Institutional Animal Care & Use Committee approvals.

### Cell culture

All reagents were from Sigma (St. Louis, MO) or Fisher (Waltham, MA) unless otherwise indicated. A431-shDsg2, A431-shGFP, HaCaT-shDsg2, and HaCaT-shGFP cells were previously established (Gift from Dr. James K. Wahl III, University of Nebraska, [15]. Cells were grown to ~70% confluency in complete DMEM with 10% FBS (Gemini Bio Products, West Sacramento, CA) and then serum starved (0.5% FBS) for 24–48 hours prior to lysis in Triton X-100 lysis buffer for Western analysis.

### Western analysis

Proteins were resolved over 5–10% SDS-PAGE (Bio-Rad Laboratories), transferred to PVDF membrane, non-specific sites blocked in 5% Carnation milk in PBS/Tween-20 and incubated in primary antibodies in 1% BSA in PBS/T-20 overnight at 4°C. Membranes were washed and incubated with HRP-conjugated secondary antibodies from Jackson Immunoresearch (West Grove, PA) and signal was detected with chemiluminescence (ECL, GE Life Sciences, Piscataway, NJ). Antibodies: 10D2 (1:100; gift from Dr. Wahl), Actin (1:100, 000; Calbiochem; Billerica, MA), Gli1 (1:1000; Cell Signaling Technology, Danvers, MA). Quantitation performed using ImageJ available at http://rsb.info.nih.gov/ij; developed by Wayne Rasband (National Institutes of Health, Bethesda, MD).

### RNA-extraction and RT-qPCR

RNA was extracted from cultured cells or skin tissues using RNAeasy kit (Qiagen; Valencia, CA) or Trizol (Life Technologies, Carlsbad, CA), respectively. DNA was removed using TURBO DNase I (Life Technologies) and cDNA was generated using High-Capacity cDNA Reverse Transcription Kit (Life Technologies). qPCR was performed on BioRad MiniOpticon with Sso Eva Green (Bio-Rad, Hercules, CA). Forward (F) and reverse (R) primers are as follows.

Mouse – Dsg2-F: GAGGAATTGAGTGCAGCACATAC,

Dsg2-R: CTTGCTTCCACCGTCAAGG;

Gli1-F: GTCCGCGCCTCTCCCACATACTA,

Gli1-R: ACGCTCGCAGGGCAGGGATAG;

Ptc1-F: GGAAGGGGCAAAGCTACAGT,

Ptc1-R: TCCACCGTAAAGGAGGCT TA;

Sox9-F: TCGGTGAAGAACGGACAAGC,

Sox9-R: TGAGATTGCCCAGAGTGCTCG;

Gapdh-F: CCCATCACCATCTTCCAGGAGCGA;

Gapdh-R: TCCACCCTTCAAGTGGCCC.

Human – DSG2-F: GAAGAGTTGAGTGCAGCACATAC,

DSG2-R: CTTGCTTCTACTGTCAAAGTCTACG;

GLI1-F: CCAACTCCACAGGCATACAGGATCCC,

GLI1-R: TCTTGGGAGTCAAATTCCTGGCTGCA;

GAPDH-F: CCCATCACCATCTTCCAGGAGCGA,

GAPDH-R: CCCCCTGCAAATGAGCCCCAG.

### Transgenic and knock-in mouse models

All animal studies were in compliance with the Institutional Animal Care & Use Committee approvals. Ptc1^+/LacZ^ knock-in reporter mice (Ptch1^tm1Mps^/J) were obtained from Jackson Labs (Bar Harbor, ME). Inv-Dsg2 mice were previously generated and described in detail [7]. All animals were maintained under ALAAC approved conditions. Ptc1^+/LacZ^ and Inv-Dsg2 mice were crossed to yield WT, Ptc1^+/LacZ^ Inv-Dsg2 and Inv-Dsg2/Ptc1^+/LacZ^ mice. Animals were sacrificed at indicated time points and tissues collected for analysis.

### DMBA-TPA skin carcinogenesis

DMBA (7,12-dimethylbenz[a]anthracene; 400 nmol in 200 μl acetone) was applied directly to shaved backskin of 6–8 week old mice under red light, and housed overnight in the dark. Subsequently, mice were treated topically, twice weekly with TPA (12-O-tetradecanoylphorbol 13-acetate; 17 nmol in 200 μl acetone) for 26 weeks. Tumors were counted and measured weekly. After 26 weeks mice were sacrificed and tumors collected for analysis.

### Histology, microscopy, and morphometric analysis

Tissues were fixed in 10% formalin, paraffin embedded, processed for histology with Hematoxylin and Eosin. Bright field images were acquired with either EVOS microscope system (Life Technologies, Carlsbad, CA) or Zeiss Axioshop microscope (Carl Zeiss Microscopy, Thornwood, NY) with SPOT Insight camera and software (HiTech Instruments, Pennsburg, PA). Epidermal measurements were performed using EVOS software. BCCs were identified in H&E sections by the following characteristics: discrete, nodular aggregations of hyperchromatic epithelial basaloid cells with prominent nuclei and scant cytoplasm; tumor area measurements were completed using ImageJ.

### BrdU incorporation assay

Mice were injected subcutaneously in the dorsal region with 5-bromo-2-deoxyuridine (50 mg/gram). After one hour, mice were sacrificed and tissue collected for processing. Direct immunofluorescence was performed using Anti-BrdU-FITC (BD Biosciences, San Jose, CA). A percentage of BrdU-positive over total epidermal basal cells was reported.

### LacZ reporter gene detection

Tissues were fixed in 4% paraformaldehyde for 20–40 minutes at room temperature and incubated overnight in 1 mg/ml X-Gal (5-bromo-4-chloro-3-indolyl-beta-D-galacto-pyranoside) at 37°C. Tissues were then processed for OCT or histology, and counterstained with Nuclear Fast Red (Rowley Biochemical, Danvers, MA).

### Immunofluorescence (IF) and immunohistochemistry (IHC)

Immunostaining was performed on formalin-fixed paraffin embedded sections as previously described [6]. IF utilized Alexa-Flour secondary antibodies (Life Technologies) with DAPI (100 ng/ml) for DNA counterstaining. IHC was completed with EnVision HRP antibody system (Dako, Carpinteria, CA). Antibodies: anti-FLAG pAb, 1:1000 (Sigma); anti-loricrin mAb, 1:1000 (Covance); anti-β-gal pAb 1:5000 (MP Biomedicals, Santa Ana, CA); 5E1 anti-Shh mAb 1:50 (DSHB, Iowa City, IA); anti-Phospho-Erk1/2 1:400 (Cell Signaling, Danvers, MA).
